# Spontaneous closure of a myopic macular hole with retinal reattachment in an eye with high myopia and staphyloma: a case report

**DOI:** 10.1186/1471-2415-14-111

**Published:** 2014-09-18

**Authors:** Jia Yu, Chunhui Jiang, Gezhi Xu

**Affiliations:** Department of Ophthalmology, Eye & ENT Hospital of Fudan University, 83 Fenyang Road, Shanghai, 200031 People’s Republic of China; Shanghai Key Laboratory of Visual Impairment and Restoration, Fudan University, Shanghai, China

**Keywords:** Myopia, Macular hole, Retinal detachment, Optical coherence tomography

## Abstract

**Background:**

Macular hole related retinal detachment is a common entity with poor surgical prognosis in highly myopic eyes. We describe the first case of spontaneous closure of a macular hole with complete retinal reattachment in a highly myopic eye with posterior staphyloma.

**Case presentation:**

A 64-year-old Chinese woman with high myopia was diagnosed as having a macular hole-related retinal detachment with vitreo-retinal traction in her right eye by optical coherence tomography. Thirty-three months later, the macular hole closed, with formation of a lamellar hole and decreased retinal detachment. Twelve months later, retinal reattachment was found to have occurred, accompanied by the development of macular retinoschisis. Fifty-four months after initial examination, the retina remained attached with a lamellar hole and retinoschisis in the macular area. The vitreo-retinal traction persisted during the follow-up period.

**Conclusion:**

As evidenced by the current case, in highly myopic eyes, the vitreoretinal traction force, which contributes to a macular hole and retinal detachment, could be partially released by the development of a lamellar hole or foveal schisis. This reduction of traction might contribute to retinal reattachment.

## Background

Macular hole related retinal detachment is a common entity in highly myopic eyes [1]. Results of surgical procedures, like pneumaticretinopexy and pars plana vitrectomy combined with other techniques, have not had favorable outcomes [2–5]. In contrast to common reports of spontaneous closure of idiopathic macular holes [6–8], reports of spontaneous closure of highly myopic macular holes have been exceedingly rare [9]. We report here the first case of spontaneous closure of a macular hole with complete retinal reattachment in a highly myopic eye.

## Case presentation

**Figure 1:**
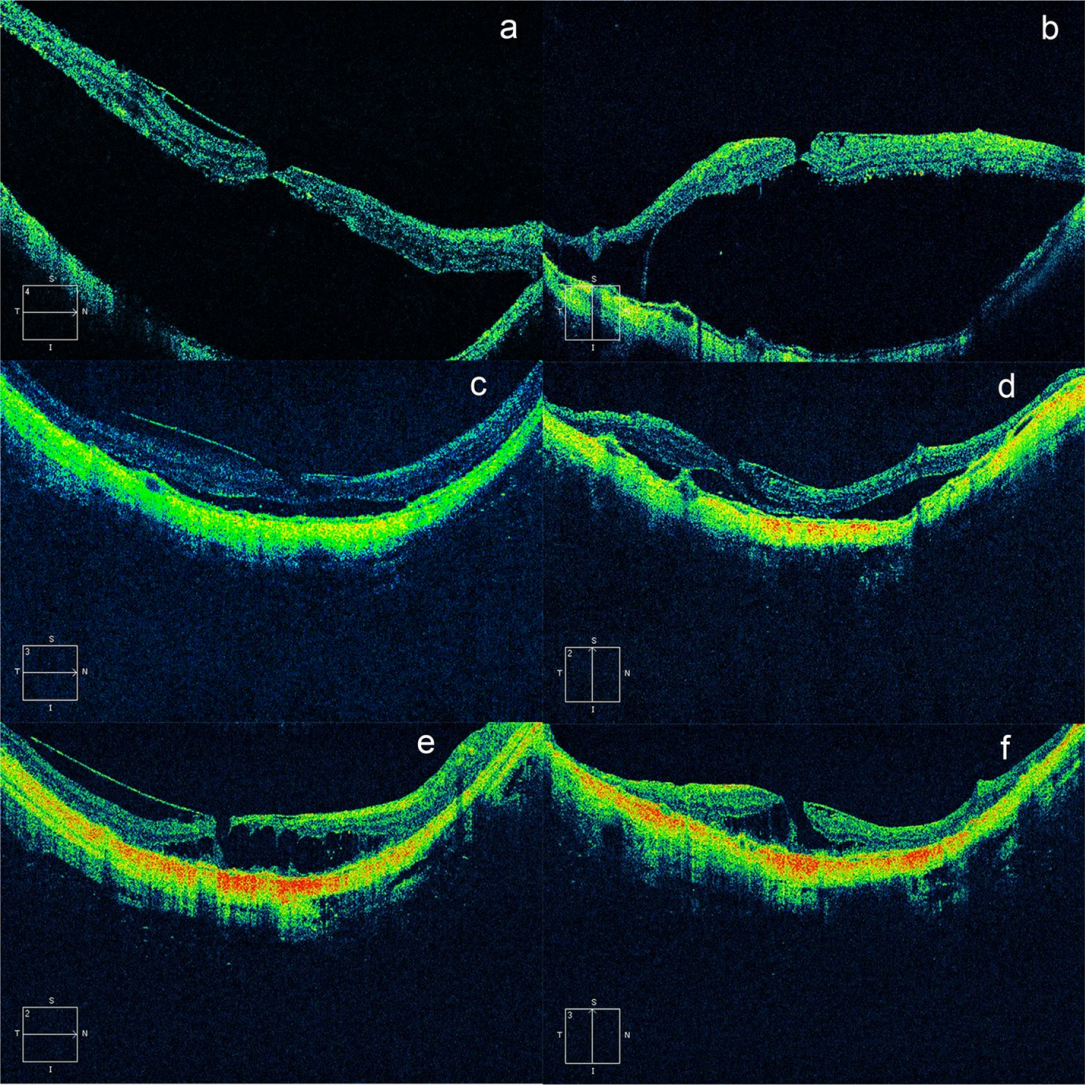
**Spontaneous closure of a macular hole and complete retinal reattachment in a highly myopic eye.** Panels **a**, **c** and **e** were horizontal scans, while panels **b**, **d**, **f**, were vertical scans. Retinal detachment and vitreoretinal traction caused by the posterior hyaloid membrane were presented **(a, b)**; a full thickness macular hole was noted accompanied by para macular schisis inferior to the macula **(b)**. The macular hole was closed (33 months later) and a lamellar hole formed with decreased retinal detachment **(c, d)**. The retina (another 21 months later) stayed completely reattached with macular retinoschisis surrounding the fovea. The posterior hyaloid was detached from the inner surface of the retina in a wider area throughout the follow-up **(e, f)**.

A 64-year-old female visited our hospital for poor vision in her right eye. Ophthalmological examination indicated her vision was counting fingers and diffuse chorioretinal atrophy at the posterior pole was observed. A B scan showed posterior staphyloma and a localized retinal detachment at the posterior pole. An A scan showed the axial length was 31.37 mm. Optical coherence tomography (OCT, Cirrus HD-OCT, Carl Zeiss Meditec, Dublin, CA, axial resolution 5 μm) revealed a full-thickness macular hole, 66 μm in diameter, accompanied by retinal detachment and vitreo-retinal traction from the posterior hyaloid membrane that was attached to the macular area (Figure 1a and b). The patient chose to be observed after reviewing the risks and benefits of surgery. Thirty-three months later, the macular hole was closed, with the formation of a lamellar hole and decreased retinal detachment (Figure 1c and d). Twelve months later, retinal reattachment was observed to be complete, accompanied by the development of macular retinoschisis. As indicated by subsequent OCT scans, such a condition remained stable, and vision remained at counting fingers until 54 months after the initial examination. The posterior hyaloid membrane was detached from the inner surface of the macula in a wider area during the follow-up (Figure 1e and f).

## Conclusion

Recently, Li et al. reported the first case of spontaneous closure of a highly myopic macular hole, which was ascribed to the release of the vitreoretinal tractional force [9]. However, in the current case, vitreo-retinal traction from the posterior hyaloid membrane was found initially and remained present throughout the follow-up, thus the release of vireoretinal traction was unlikely. In this case, closure of the hole might have happened first, albeit the reason not fully being understood. The small size of the macular hole in the current case might be one cause. It has been reported that in both emmetropic and highly myopic eyes, small macular holes were more likely to be closed after surgical treatment [10, 11]. After closure, however, the retinal detachment was reduced with the reabsorption of subretinal fluid by the retinal pigment epithelium (RPE) pump, though this might be inefficient because of the degeneration and atrophy of the RPE and choroid. However, the vitreo-retinal traction that was still present prevented the retina from laying down and, consequently, these two forces might actually lead to tissue dehiscence; i.e., the development of a lamellar hole and macular schisis. In highly myopic eyes, traction forces caused by the stretching of the eyeball and relative shortening of the retina, as well as vitreo-retinal traction by the posterior hyaloid membrane, contribute to the development of many macular disorders [12–14]. With the formation of a lamellar hole, the retina might be elongated by the dehiscence of the tissues, resulting in reduction of the traction force. This could be partially supported by the findings that a lamellar hole is a relatively stable condition in highly myopic eyes [15]. Also, the subsequent development of retinal schisis might further reduce the traction force in a similar way. As indicated in Figure 1e and f, at the final follow-up, the retina was reattached with the presence of posterior hyaloid traction; therefore, the traction force was not released but was reduced by the development of a lamellar hole and foveal schisis.

It remains unclear whether surgical intervention could improve the visual outcomes. As evidenced by the current case, nevertheless, spontaneous closure of a macular hole and complete retinal reattachment can occur in a myopic eye. Future study with more cases might further improve our knowledge of this entity.

## Consent

Written informed consent was obtained from the patient for publication of this case report and any accompanying images. A copy of the written consent is available for review by the Editor of this journal.

## Authors’ information

Dr. Chunhui Jiang conducts one project of Shanghai Committee of Science and Technology, and Dr. Gezhi Xu conducts 2 projects supported by National Key Basic Research Program of China and Shanghai Key Laboratory of Visual Impairment and Restoration, Fudan University, Shanghai, China, respectively.

## Abbreviations

OCT: Optical coherence tomography
RPE: Retinal pigment epithelium.
